# Uncertainty-Based Fusion Method for Structural Modal Parameter Identification

**DOI:** 10.3390/s25144397

**Published:** 2025-07-14

**Authors:** Xiaoteng Liu, Zirui Dong, Hongxia Ji, Zhenjiang Yue, Jie Kang

**Affiliations:** 1 College of Astronautics, Nanjing University of Aeronautics and Astronautics, Nanjing 211106, China; liuxiaoteng@nuaa.edu.cn (X.L.); kangjie@nuaa.edu.cn (J.K.); 2 Shanghai Institute of Spacecraft Equipment, Shanghai 201109, China; yuezhuhuakai@163.com (Z.D.); hongling1977@126.com (H.J.); 3 Intelligent Science and Technology Academy of CASIC, Beijing 100043, China

**Keywords:** operational modal analysis, uncertainty quantification, data fusion, AutoRegressive model, Left-Matrix Fraction model

## Abstract

The structural modal parameter identification method can be classified into time-domain and frequency-domain methods. Practically, two types of methods are characterized by different advantages, and the estimated modal parameters are always subjected to statistical uncertainties due to measurement noise. In this work, an uncertainty-based fusion method for structural mode identification is proposed to merge the advantages of different methods. The extensively applied time-domain AutoRegressive (AR) and frequency-domain Left-Matrix Fraction (LMF) models are expressed in a unified parametric model. With this unified model, a generalized framework is developed to identify the modal parameters of structures and compute variances associated with modal parameter estimates. The final modal parameter estimates are computed as the inverse-variance weighted sum of the results identified from different methods. A numerical and an experimental example demonstrate that the proposed method can obtain reliable modal parameter estimates, substantially mitigating the occurrence of extremely large estimation errors. Furthermore, the fusion method demonstrates enhanced identification capabilities, effectively reducing the likelihood of missing structural modes.

## 1. Introduction

Operational modal analysis (OMA) is extensively applied in structural engineering to extract modal parameters (i.e., modal frequencies, damping ratios, and mode shape vectors) of structures from dynamic responses [1,2,3]. The OMA techniques can be categorized into time- and frequency-domain methods [2,3,4,5].

Time-domain methods typically employ parametric time series models, including the AutoRegressive (AR), AutoRegressive and Moving Average (ARMA), and state space model [2,3,6]. The AR model expresses the dynamic responses at the current time instant as a linear combination of the responses at past instants [7,8]. The modal parameters are identified by solving a matrix polynomial eigenvalue problem derived from model coefficients. The OMA approach based on the state space model is referred to as the Stochastic Subspace Identification (SSI) method, which can be classified into two branches, i.e., the COVariance-driven SSI (SSI-COV) and DATA-driven SSI (SSI-DATA) versions [1,2,9]. In the SSI-COV method, a Hankel matrix is constructed from the correlation functions of random responses, and the system matrices of the state space model are derived by the Singular Value Decomposition (SVD) technique. Alternatively, the DATA-SSI method identifies modal parameters by projecting future data onto the subspace spanned by past data. Alternatively, frequency-domain OMA methods utilize parameterized models, such as the Left-Matrix Fraction (LMF), the Right-Matrix Fraction (RMF), and the common-denominator model, to analyze the Power Spectral Density (PSD) functions of responses [4,10,11]. Modal parameters are derived by solving a matrix polynomial eigenvalue problem constructed from model coefficients analogous to time-domain techniques. Although frequency-domain methods use the estimated PSD functions of responses as input data, their accuracy depends on the quality of the estimated PSDs [5]. The parameters in Welch’s method for estimating PSDs can influence the results of OMA. In addition, the LMF method exhibits superior performance in identifying structural modes with high damping ratios, and the identification capability can be improved by applying a limited frequency band [3,11]. Unlike AR-based methods that process time series data with all $N_{o}$ channels directly, the LMF method requires computing PSD functions, resulting in increased computational complexity compared to their time-domain counterparts.

In practice, the modal parameters identified by OMA techniques are always subject to statistical uncertainties arising from various sources, such as finite data length, measurement noise and numerical errors [12,13,14,15]. In the context of OMA, furthermore, the ambient excitation acting on the structure is assumed to be white noise sequences and is not involved in parametric models, so the uncertainties are much larger compared to those modes’ identification methods with known excitation [16]. Over the past decade, the uncertainty quantification problem in OMA has attracted much attention, and a series of methods have been proposed. In the frequency domain, Pintelon et al. [12] first proposed an uncertainty quantification method for modal parameter identification based on the Common Denominator Model (CDM). Based on this foundational concept, Troyer et al. [17] further developed an uncertainty quantification approach for the poly-reference Least-Squares Complex Frequency (pLSCF) algorithm. Steffensen et al. [18] calculated the variance matrix of complete modal parameters estimated by the RMF model. In the time domain, Reynders et al. [9,19] computed the variance of modal parameters derived via the SSI algorithm, and Mellinger et al. [20] proposed a unified framework for uncertainty quantification by integrating four distinct subspace identification methods. Xu et al. [21] quantified the uncertainty in modal parameters utilizing the nonparametric random decrement technique. Alternatively, Yang et al. [8] introduced a Bayesian system identification method using the AR model, and Kang et al. [16] further developed a variance computation method using the time-dependent ARMA (TARMA) model for time-varying structure identification. Among these methods, the first-order perturbation approach is extensively applied to compute Jacobian matrices relating model coefficients to modal parameters, under the assumptions that the Signal-to-Noise ratio (SNR) of the measured data is sufficiently high and the modal parameters follow Gaussian distributions [12,13,16].

To comprehensively exploit the information embedded within measured data, the integration of time- and frequency-domain methods has been developed in some recent studies. For instance, Brandt [22] developed a signal processing framework for OMA that unifies time- and frequency-domain approaches, offering an effective means to simultaneously estimate both correlation functions and spectral densities. Kang et al. [23] advanced a unified framework for modal identification and uncertainty quantification based on four types of transmissibility functions. In Ref. [24], a multi-step modal identification method based on joint time-frequency analysis was presented, which could be applied to non-stationary vibration signal processing. Volkmar et al. [25] developed a unified automated modal analysis approach that integrates the SSI and pLSCF methods, which could be applied to both Experimental Modal Analysis (EMA) and OMA. Among these studies mentioned above, the existing fusion methods primarily focus on either constructing unified frameworks applicable to both time- and frequency-domain identification or developing multi-step strategies to enhance the modal analysis performance. However, there remains limited exploration of modal identification methods that explicitly fuse identification results derived from distinct models or methodologies. In the context of signal processing, the variance-based data fusion method has been extensively researched and applied to merge information measured from multiple sensors for deriving more reliable results [26,27]. Huang et al. [28] proposed a fusion method of multiple inertial measurement units based on measurement noise variance estimation. Qi et al. [29,30] designed the local and five weighted fused robust time-varying Kalman predictors, and the prediction error variances achieve the corresponding minimal upper bounds for all admissible uncertainties of noise variances. George et al. [31] compared two data fusion methods, i.e., the variance-based fusion and centralized Kalman filter, for target tracking. The inverse-variance weighted fusion method can achieve the results of minimal variances, which is very important for many tasks in engineering, such as damage detection, object recognition, and vehicle navigation.

Following these prior researches, this work aims to propose an uncertainty-based fusion method to identify the modal parameters of time-invariant linear structures. A parametric model is first formulated to integrate the LMF and AR models. With this unified parametric model, a generalized identification approach based on the Negative Log-Likelihood Function (NLLF) is developed under the assumption of Gaussian innovation sequences. Subsequently, the uncertainties associated with the modal parameter estimates are quantified using the first-order perturbation theory. Finally, the identification results derived from individual methods are fused through an uncertainty-based fusion scheme, thereby generating more reliable and robust modal parameter estimates.

The remainder of this paper is organized as follows. In Section 2, the detailed formulas and implementation procedures of the proposed method are presented. A numerical model and an experimental beam are used to validate the method proposed in Section 3. Finally, some important discussions and conclusions about the proposed fusion method are presented in Section 4 and Section 5, respectively.

## 2. Methods

### 2.1. AR and LMF Model

For a time-invariant linear structure, the time-domain dynamic responses subjected to broad-band noise excitation can be represented by an AR model [3]

(1) $$x\left[t\right]+\sum_{j=1}^{n_{1}}D_{j}x\left[t-j\right]=e\left[t\right],\;e\left[t\right]∼\mathrm{NID}\left(0,\Sigma_{e}\right)$$

where $x\in R^{N_{o}\times 1}$ is the response vector measured by $N_{o}$ sensors; $D_{j}$ is the coefficient matrix; $n_{1}$ is the AR model order; *t* is the time instant; $e\left[t\right]$ is the innovation sequence, which is normally distributed and independent with respect to different time instants; and $\mathrm{NID}\left(0,\Sigma_{e}\right)$ designates the normal independent distribution with zero vector and variance matrix $\Sigma_{e}$.

In the frequency domain, the PSD functions of the responses can be modeled by the LMF description [10]

(2) $$\left(I_{N_{o}}+\sum_{j=1}^{n_{2}}N_{j}e^{-i\omega_{k}T_{s}j}\right)G\left[\omega_{k}\right]=\sum_{i=0}^{n_{3}}L_{i}e^{-i\omega_{k}T_{s}i}+E\left[\omega_{k}\right]$$

where $I_{N_{o}}$ is the unity matrix with dimension of $N_{o}\times N_{o}$; $G\in C^{N_{o}\times N_{i}}$ is the PSD matrix of responses; $n_{2}$ and $n_{3}$ are the denominator and numerator orders, respectively; $N_{j}$ and $L_{i}$ are denominator and numerator coefficient matrices, respectively; $T_{s}$ is the sampling interval; $\omega_{k}$ is the *k*th frequency line in unit rad/s; and E is the residual matrix. The method for estimating the coefficients of the LMF model is provided in Appendix A.

**Remark 1.**  *The complete PSD matrix of the responses measured by $N_{o}$ sensors is of dimension $N_{o}\times N_{o}$. However, a sub-block of the complete PSD matrix is usually used in LMF models to reduce the computational complexity. In this work, only a single column of the complete PSD matrix is used in LMF to identify the modal parameters of a structure, and then the results are fused to generate more reliable modal parameter estimates. In this scenario, $N_{i}=1$.*

**Remark 2.**  *If the original PSDs are used in the LMF model, the model order will be twice the true order of the underlying structure, which may result in numerical instability. Therefore, the Positive Spectrum Functions (PSFs) [32,33,34], instead of the original PSDs, are adopted to construct the LMF model in this work. The PSF can be computed using the following procedure: first, the PSD function is transformed to the time-domain correlation function using the inverse Fourier transform; then, the negative part of correlation function is set to zero; finally, the PSF is derived by applying Fourier transform to the reshaped correlation function.*

**Remark 3.**  *The model orders $n_{1}$, $n_{2}$ and $n_{3}$ can be determined using some fitness criteria, e.g., the Akaike Information Criterion (AIC) and Bayesian Information Criterion (BIC). In this work, the BIC is adopted for this purpose. The BIC would exhibit a sharp descending trend first and then a plateau or a slight ascending trend for increasing model orders. Therefore, the model orders can be determined when the BIC shows no abrupt changes.*

### 2.2. Unified Model and Model Coefficients Estimation

The time-domain AR and frequency-domain LMF models given in Equations (1) and (2) can be unified using a generalized parametric model [3,23]

(3) $$y\left[p\right]+\sum_{j=1}^{n}A_{j}\left(q^{-j}y\left[p\right]\right)=\sum_{j=0}^{m}b_{j}q^{-j}+e\left[p\right],\;e\left[p\right]∼\mathrm{NID}\left(0,\Sigma_{e}\right)$$

where y is the time-domain response or frequency-domain PSF vector; *p* is the ppth frequency line in unit rad/s for PSFs or time instant for time-domain responses, and p=0,1,…,N−1 with *N* designating the length of data; *n* and *m* are model orders; $A_{j}$ is the *j*th denominator coefficient matrix; and $b_{j}$ is the numerator coefficients. In the frequency domain, the operator $q^{-1}y\left[p\right]=e^{-i\omega_{p}T_{s}}y\left[p\right]$, while in time domain $q^{-1}y\left[p\right]=y\left[p-1\right]$; $e\left[p\right]$ is the zero-mean innovation sequence vector with variance matrix $\Sigma_{e}$; and $e\left[p_{1}\right]$ and $e\left[p_{2}\right]$ are independent when $p_{1}\neq p_{2}$.

The unified model in Equation (3) can be written compactly as

(4) $$y\left[p\right]=-\Theta \psi \left[p\right]+e\left[p\right]$$

where

$$\begin{array}{cc} \Theta & =\left[\begin{array}{cccccc} A_{n} & \ldots & A_{1} & -b_{m} & \ldots & -b_{0} \end{array}\right]\\ \psi [p] & ={\left[\begin{array}{cccccc} q^{-n}y^{T}\left[p\right] & \ldots & q^{-1}y^{T}\left[p\right] & q^{-m} & \ldots & 1 \end{array}\right]}^{T} \end{array}$$

where the superscript T stands for the transpose of a matrix.

For the unified model in Equation (4), the likelihood function of the observed data given parameters Θ and $\Sigma_{e}$ is

(5) $$P\left(y_{0:N-1};\Theta ,\Sigma_{e}\right)=\prod_{p=0}^{N-1}\frac{1}{{\left(2\pi \right)}^{N_{o}/2}{\left|\Sigma_{e}\right|}^{1/2}}e^{-\frac{1}{2}{\left(y\left[p\right]+\Theta \psi \left[p\right]\right)}^{H}\Sigma_{e}^{-1}\left(y\left[p\right]+\Theta \psi \left[p\right]\right)}$$

with $\left|\cdot \right|$ representing the determinant of a matrix or the absolute value of a number, $y_{0:N-1}$ is the set of all data points, and the superscript −1 designates the inverse matrix. The NLLF can be expressed as

(6) $$\begin{array}{cc} L\left(y_{0:N-1};\Theta ,\Sigma_{e}\right)= & -\ln\left(P\left(y_{0:N-1};\Theta ,\Sigma_{e}\right)\right)\\ = & c+\frac{N}{2}\ln\left|\Sigma_{e}\right|+\frac{1}{2}\sum_{p=0}^{N-1}{\left(y\left[p\right]+\Theta \psi \left[p\right]\right)}^{H}\Sigma_{e}^{-1}\left(y\left[p\right]+\Theta \psi \left[p\right]\right) \end{array}$$

where $\ln\left(\cdot \right)$ is the natural logarithm of the argument and *c* is a constant; the superscript H means the conjugate transpose of a matrix.

In Bayesian inference, the prior knowledge of the unknown parameters can be introduced to update the posterior distribution of parameters using Bayes’s theorem. If no prior knowledge is available, the posterior distribution of the parameters is proportional to the likelihood function. In this scenario, the Maximum Likelihood Estimate (MLE) of unknown parameters can be estimated by letting the corresponding partial differentials be zeros (the formulas $\frac{\partial \left|A\right|}{A}=\left|A\right|A^{-T}$ and $\frac{\partial a^{H}A^{-1}b}{\partial A}=-A^{-T}a^{*}b^{T}A^{-T}$ are used to derive the second row):

(7) $$\begin{array}{cc} \frac{\partial L\left(y_{0:N-1};\Theta ,\Sigma_{e}\right)}{\partial \Theta } & =\sum_{p=0}^{N-1}\Sigma_{e}^{-1}\left(\Theta \psi \left[p\right)]\psi^{H}\left[p\right)]+y\left[p\right]\psi^{H}\left[p\right)]\right)=0\\ \frac{\partial L\left(y_{0:N-1};\Theta ,\Sigma_{e}\right)}{\partial \Sigma_{e}} & =\frac{N}{2}\Sigma_{e}^{-T}-\frac{1}{2}\sum_{p=0}^{N-1}\left(\Sigma_{e}^{-T}e^{*}\left[p\right]e^{T}\left[p\right]\Sigma_{e}^{-T}\right)=0 \end{array}$$

with the superscript * representing the conjugate of the argument. Noting that $\Sigma_{e}$ is a conjugate symmetric matrix, the solution is

(8) $$\begin{array}{cc} \hat{\Theta } & =-\left(\sum_{p=0}^{N-1}ℜ\left(y\left[p\right]\psi^{H}\left[p\right]\right)\right){\left(\sum_{p=0}^{N-1}ℜ\left(\psi \left[p\right]\psi^{H}\left[p\right]\right)\right)}^{-1}\\ {\hat{\Sigma }}_{e} & =\frac{1}{N}\sum_{p=0}^{N-1}\hat{e}\left[p\right]{\hat{e}}^{H}\left[p\right] \end{array}$$

where $ℜ\left(\cdot \right)$ is the real part of the argument and the estimated innovation sequence is

(9) $$\hat{e}\left[p\right]=y\left[p\right]+\hat{\Theta }\Psi \left[p\right]$$

At the vicinity of $\hat{\Theta }$, the conditional NLLF with estimated ${\hat{\Sigma }}_{e}$ can be approximated using the second-order Taylor expansion [16]

(10) $$L\left(y_{0:N-1};\Theta ,{\hat{\Sigma }}_{e}\right)\approx L\left(y_{0:N-1};\hat{\Theta },{\hat{\Sigma }}_{e}\right)+\frac{1}{2}{\left(\theta -\hat{\theta }\right)}^{T}{\left.\frac{\partial^{2}L\left(y_{0:N-1};\Theta ,{\hat{\Sigma }}_{e}\right)}{\partial \theta \partial \theta^{T}}\right|}_{\theta =\hat{\theta }}\left(\theta -\hat{\theta }\right)$$

where $\theta =\mathrm{vec}\left(\Theta \right)$ with $\mathrm{vec}\left(\cdot \right)$ designating the vectorization operator, and ${\left.\cdot \right|}_{\theta =\hat{\theta }}$ stands for the value at $\theta =\hat{\theta }$.

With no prior knowledge of unknown parameters, the posterior distribution of unknown parameters can be expressed as

(11) $$\begin{array}{cc} P\left(\Theta ;y_{0:N-1},{\hat{\Sigma }}_{e}\right) & \propto \exp\left(-L\left(y_{0:N-1};\Theta ,{\hat{\Sigma }}_{e}\right)\right)\\ \; & \approx P\left(y_{0:N-1};\hat{\Theta },{\hat{\Sigma }}_{e}\right)\exp\left(-\frac{1}{2}{\left(\theta -\hat{\theta }\right)}^{T}{\left.\frac{\partial^{2}L\left(y_{0:N-1};\Theta ,{\hat{\Sigma }}_{e}\right)}{\partial \theta \partial \theta^{T}}\right|}_{\theta =\hat{\theta }}\left(\theta -\hat{\theta }\right)\right) \end{array}$$

where ∝ means "proportional to" and $\exp\left(\cdot \right)$ is the exponential of the argument. Therefore, the posterior distribution of the coefficients θ at the vicinity of the MLEs can be approximated as a Gaussian distribution, and hence the variance matrix of θ can be estimated from the inverse Hessian matrix of NLLF

(12) $${\hat{\Sigma }}_{\theta }={\left.\frac{\partial^{2}L\left(y_{0:N-1};\Theta ,{\hat{\Sigma }}_{e}\right)}{\partial \theta \partial \theta^{T}}\right|}_{\theta =\hat{\theta }}={\left(\sum_{p=0}^{N-1}ℜ\left(\psi \left[p\right]\psi^{H}\left[p\right]\right)\right)}^{-1}⊗ℜ\left({\hat{\Sigma }}_{e}\right)$$

with ⊗ designating the Kronecker product.

### 2.3. Modal Parameter Identification

For the unified model in Equation (3), the signal y can be adopted as time-domain responses or the PSFs of responses. The system poles can be derived by solving the matrix polynomial eigenvalue problem

(13) $$\left(I_{N_{o}}+\sum_{j=1}^{n}A_{j}\lambda_{m}^{j}\right)\phi_{m}=0$$

where $\lambda_{m}$ and $\phi_{m}$ are the *m*th eigenvalue and corresponding eigenvector of the underlying system. This eigenvalue problem can be simplified by using the eigenvalue decomposition

(14) $$\left(\Gamma -\lambda_{m}I_{nN_{o}}\right)\varphi_{m}=0$$

with

$$\Gamma =\left[\begin{array}{ccccc} 0 & I_{N_{o}} & 0 & \ldots & 0\\ 0 & 0 & I_{N_{o}} & \ldots & 0\\ \vdots & \vdots & \vdots & \ddots & \vdots \\ -A_{n} & -A_{n-1} & -A_{n-2} & \ldots & -A_{1} \end{array}\right],\;\varphi_{m}=\left[\begin{array}{c} \lambda_{m}^{-n}\phi_{m}\\ \lambda_{m}^{-n+1}\phi_{m}\\ \vdots \\ \lambda_{m}^{-1}\phi_{m} \end{array}\right]$$

The modal frequency and damping ratio of the *m*th mode of the underlying structure are

(15) $$f_{nm}=\frac{\left|\ln\lambda_{m}\right|}{2\pi T_{s}}\mathrm{Hz},\;\xi_{m}=-\frac{ℜ\left(\ln\lambda_{m}\right)}{\left|\ln\lambda_{m}\right|}$$

The mode shape vector can be estimated from the first $N_{o}$ entries of $\varphi_{m}$, denoted as ${\bar{\varphi }}_{m}$. To obtain real mode shape vectors, the selected *k*th component of ${\bar{\varphi }}_{m}$, ${\bar{\varphi }}_{m,k}$, is first scaled to unity, and then the real part is adopted as the estimated mode shape vector.

(16) $$\phi_{m}=ℜ\left(\frac{{\bar{\varphi }}_{m}}{{\bar{\varphi }}_{m,k}}\right)$$

It should be noted that $nN_{o}$ eigenvalues can be derived for the companion matrix Γ, which is much larger than the number of structural modes. Some criteria associated with eigenvalues and modal parameters are used to remove the spurious modes included in the identification results.

(17) $$ℜ\left(\lambda_{m}\right)\geq 0;\;\xi_{m}\geq \xi_{\max};\;ℑ\left(\lambda_{m}\right)=0;\;∄\lambda_{m^{\prime }},\;s.t.\;\lambda_{m^{\prime }}=\lambda_{m}^{*}\left(m^{\prime }\neq m\right)$$

where ∄ is the ’there does not exist’ symbol and $ℑ\left(\cdot \right)$ is the imaginary part of a complex number. In Equation (17), the first criterion removes the identified modes with negative damping ratios; the second deletes those with damping ratios larger than $\xi_{\max}$ (usually 10–20%); the third removes the real eigenvalues; and the fourth requires the structural mode to be characterized as conjugate eigenvalues.

### 2.4. Uncertainty of Modal Parameters

In practice, the identified modal parameters with a specified model structure are random variables due to random excitation and measurement noise, which can be approximated as multivariate normally distributed variables according to the Central Limit Theorem. As illustrated in Section 2.3, the variance of modal parameters can be estimated using the following procedure: first estimate the variance of coefficients from Equation (12), then compute the variance of the eigenvalues and eigenvectors of Γ, and finally compute the variance of modal parameters. Along with this idea, the variance of modal parameters is quantified using the first-order perturbation method reported in Refs. [9,12,23].

The perturbations of vectorized Γ can be obtained by rearranging the perturbations of Θ

(18) $$\Delta \gamma =P\Delta \theta_{A}$$

where Δ represents the sufficiently small perturbation; $\gamma =\mathrm{vec}\left(\Gamma \right)$, $\theta_{A}=\mathrm{vec}\left(\Theta_{A}\right)$, and $\Theta_{A}$ designate denominator coefficients; and $P\in R^{n^{2}N_{o}^{2}\times nN_{o}^{2}}$ is a permutation matrix satisfying $\gamma =P\theta_{A}$.

The perturbations of Γ propagate to its *m*th eigenvalue and eigenvector by [8,13]

(19) $$\Delta \lambda_{m}=S_{\lambda_{m},\Gamma }\Delta \gamma ,\;\Delta \varphi_{m}=S_{\varphi_{m},\Gamma }\Delta \gamma$$

where $S_{\lambda_{m},\Gamma }$ and $S_{\varphi_{m},\Gamma }$ are expressed as

(20) $$S_{\lambda_{m},\Gamma }=\frac{\varphi_{m}^{T}⊗\psi_{m}^{H}}{\psi_{m}^{H}\varphi_{m}},\;S_{\varphi_{m},\Gamma }=\varphi_{m}^{T}⊗\left[{\left(\lambda_{m}I_{nN_{o}}-\Gamma \right)}^{+}\left(I_{nN_{o}}-\frac{\varphi_{m}\psi_{m}^{H}}{\psi_{m}^{H}\varphi_{m}}\right)\right]$$

with $\psi_{m}$ designating the *m*th left eigenvector of Γ that satisfies $\psi_{m}^{H}\Gamma =\lambda_{m}\psi_{m}^{H}$, the superscript + representing the pseudo inverse of a matrix.

The perturbations of the modal frequency and the damping ratio resulting from $\Delta \lambda_{m}$ are

(21) $$\Delta \eta_{m}=\left[\begin{array}{c} \Delta f_{nm}\\ \Delta \xi_{m} \end{array}\right]=S_{\eta_{m},\lambda_{m}}\left[\begin{array}{c} \Delta ℜ\left(\lambda_{m}\right)\\ \Delta ℑ\left(\lambda_{m}\right) \end{array}\right]$$

where the sensitivity matrix $S_{\eta_{m},\lambda_{m}}$ is [8,13]

(22) $$\begin{array}{cc} S_{\eta_{m},\lambda_{m}}= & \left[\begin{array}{cc} S_{f_{nm},\lambda_{m}^{ℜ}} & S_{f_{nm},\lambda_{m}^{ℑ}}\\ S_{\xi_{m},\lambda_{m}^{ℜ}} & S_{\xi_{m},\lambda_{m}^{ℑ}} \end{array}\right]\\ = & \frac{1}{T_{s}{\left|\lambda_{m}\right|}^{2}\left|\mu_{m}\right|}\left[\begin{array}{cc} \frac{ℜ\left(\mu_{m}\right)}{2\pi } & \frac{ℑ\left(\mu_{m}\right)}{2\pi }\\ -\frac{ℑ{\left(\mu_{m}\right)}^{2}}{{\left|\mu_{m}\right|}^{2}} & \frac{ℜ\left(\mu_{m}\right)ℑ\left(\mu_{m}\right)}{{\left|\mu_{m}\right|}^{2}} \end{array}\right]\left[\begin{array}{cc} ℜ\left(\lambda_{m}\right) & ℑ\left(\lambda_{m}\right)\\ -ℑ\left(\lambda_{m}\right) & ℜ\left(\lambda_{m}\right) \end{array}\right] \end{array}$$

where $\mu_{m}$ is the *m*th eigenvalue of the underlying structure in the continuous-time domain, which is $\mu_{m}=\frac{\ln\lambda_{m}}{T_{s}}$.

The sensitivity matrix of ${\bar{\varphi }}_{m}$ with respect to Γ can be derived from the first $N_{o}$ rows of $S_{\varphi_{m},\Gamma }$

(23) $$S_{{\bar{\varphi }}_{m},\Gamma }={\left[S_{\varphi_{m},\Gamma }\right]}_{1:N_{o},:}$$

The sensitivity matrices of modal parameters with respect to Γ are [12,16]

(24) $$S_{\eta_{m},\Gamma }=S_{\eta_{m},\lambda_{m}}S_{\lambda_{m},\Gamma },\;S_{\phi_{m},\Gamma }=ℜ\left(S_{{\bar{\phi }}_{m},{\bar{\varphi }}_{m}}S_{{\bar{\varphi }}_{m},\Gamma }\right)$$

with

$$S_{{\bar{\phi }}_{m},{\bar{\varphi }}_{m}}=\frac{1}{{\bar{\varphi }}_{m,k}}\left(I_{N_{o}}-\left[\begin{array}{ccc} 0_{N_{o}\times \left(k-1\right)} & \frac{{\bar{\varphi }}_{m}}{{\bar{\varphi }}_{m,k}} & 0_{N_{o}\times N_{o}} \end{array}\right]\right)$$

Therefore, the variance of modal parameters can be expressed as

(25) $$\Sigma_{\eta_{m}}=S_{\eta_{m},\Gamma }P{\hat{\Sigma }}_{\theta_{A}}P^{T}S_{\eta_{m},\Gamma }^{T},\;\Sigma_{\phi_{m}}=S_{\phi_{m},\Gamma }P{\hat{\Sigma }}_{\theta_{A}}P^{T}S_{\phi_{m},\Gamma }^{T}$$

where ${\hat{\Sigma }}_{\theta_{A}}$ is the variance matrix of $\theta_{A}$.

### 2.5. Identification Results Fusion

The modal parameters can be identified by distinct methods in the time and frequency domains, and in the frequency domain, different submatrices of the PSF matrix can also be used for the same purpose. The modal parameters identified from distinct methods or sub-matrices of the PSF matrix can be fused using the inverse-variance weighting approach [35]

(26) $$\bar{q}=\sum_{j=1}^{K}w_{j}q_{j}$$

where *q* can bethe modal frequency, the damping ratio or mode shape vector; $q_{j}$ is the quantity identified by the *j*th method or data set, and the variance of $q_{j}$ is $\Sigma_{j}$; $w_{j}$ is the corresponding weight; and *K* is the number of identified modal parameter sets. Assuming that the modal parameters identified from different models or data sets are independent, the weight $w_{j}$ can be derived by minimizing the variance of $\bar{q}$

(27) $$\begin{array}{cc} \min_{w_{1}\to w_{K}}:\Sigma_{\bar{q}} & =g\left(w_{1},w_{2},\ldots ,w_{K}\right)=\sum_{j=1}^{K}w_{j}^{2}\Sigma_{j}\\ s.t.\;h\left(w_{1},w_{2},\ldots ,w_{K}\right) & =\sum_{j=1}^{K}w_{j}-1=0\\ w_{j} & \geq 0,\;\mathrm{for}\;j=1,2,\ldots ,K \end{array}$$

Using the Lagrange multiplier method, the weights can be derived by solving

(28) $$\left\{\begin{array}{c} \frac{\partial g}{\partial w_{j}}=\mu \frac{\partial h}{\partial w_{j}},\;j=1,2,\ldots ,K\\ h\left(w_{1},w_{2},\ldots ,w_{K}\right)=0 \end{array}\right.$$

where μ is the Lagrange multiplier. Therefore, the solution for the weight $w_{j}$ is

(29) $$w_{j}=\frac{\prod_{k=1,k\neq j}^{K}\Sigma_{k}}{\sum_{j=1}^{K}\prod_{k=1,k\neq j}^{K}\Sigma_{k}}$$

### 2.6. Implementation Procedure of the Proposed Method

The implementation procedure of the proposed fusion method is detailed as follows.

Step 1 Measure the dynamic responses, $x\left[t\right]$, of the structure, and the sampling interval is denoted as $T_{s}$.
Step 2 Determine the model order *n* of the AR model using the BIC criterion.
Step 3 Compute coefficients ${\hat{\Theta }}_{\mathrm{AR}}$ and ${\hat{\Sigma }}_{\theta }^{\mathrm{AR}}$ of the AR model using Equations (8) and (12).
Step 4 Identify modal parameters $f_{nm}^{\mathrm{AR}}$, $\xi_{m}^{\mathrm{AR}}$, $\phi_{m}^{\mathrm{AR}}$ through Equations (14)–(16).
Step 5 Compute the variance of modal parameters $\Sigma_{\eta_{m}}^{\mathrm{AR}}$, $\Sigma_{\phi_{m}}^{\mathrm{AR}}$ using Equation (20) and Equations (22)–(25).
Step 6 Compute the PSF matrix of $x\left[t\right]$, and select $n_{c}$ columns of the PSF matrix.
Step 7 Determine the model orders *n* and *m* of the LMF model using the BIC criterion with a typical column of PSF matrix.
Step 8 For the *j*th column selected in Step 6, compute coefficients ${\hat{\Theta }}_{\mathrm{LMF}\left(j\right)}$ and ${\hat{\Sigma }}_{\theta }^{\mathrm{LMF}\left(j\right)}$ of the LMF model using Equations (8) and (12).
Step 9 Identify modal parameters $f_{nm}^{\mathrm{LMF}\left(j\right)}$, $\xi_{m}^{\mathrm{LMF}\left(j\right)}$, $\phi_{m}^{\mathrm{LMF}\left(j\right)}$ from ${\hat{\Theta }}_{\mathrm{LMF}\left(j\right)}$ through Equations (14)–(16).
Step 10 Compute the variance of the modal parameters $\Sigma_{\eta_{m}}^{\mathrm{LMF}\left(j\right)}$ and $\Sigma_{\phi_{m}}^{\mathrm{LMF}\left(j\right)}$ from ${\hat{\Sigma }}_{\theta }^{\mathrm{LMF}\left(j\right)}$ using Equation (20) and Equations (22)–(25).
Step 11 Fuse the modal parameter estimates derived by AR model (e.g., $f_{nm}^{\mathrm{AR}}$, $\xi_{m}^{\mathrm{AR}}$, $\phi_{m}^{\mathrm{AR}}$) and LMF models (e.g., $f_{nm}^{\mathrm{LMF}\left(j\right)}$, $\xi_{m}^{\mathrm{LMF}\left(j\right)}$, $\phi_{m}^{\mathrm{LMF}\left(j\right)}$) using Equation (29), where $j=1,2,\ldots ,n_{c}$.

## 3. Case Studies

### 3.1. Four-Story Shear Frame Model

**Figure 1:**
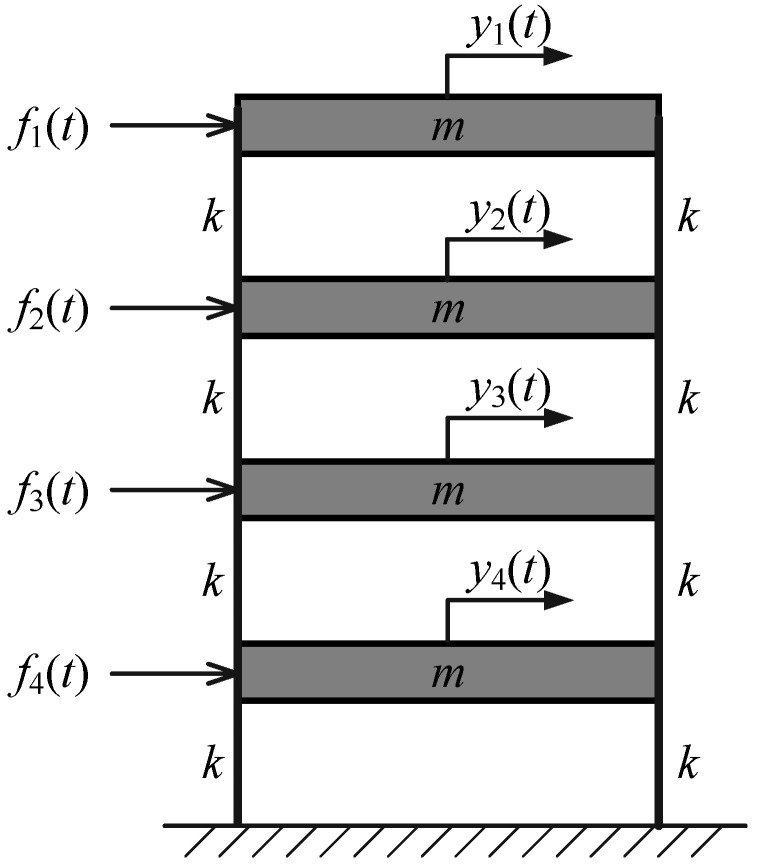
The 4-DOF shear frame model.

A four-degree-of-freedom (4-DOF) shear frame, as shown in Figure 1, is used to validate the proposed method. The mass of each story is 2 kg and the shear stiffness of each column is 2500 N/m. In this model, the proportional damping matrix $C=M+10^{-4}K$ is adopted, with M and K designating the mass and stiffness matrices, respectively. The theoretical modal parameters of the frame model are computed from M, K and C and used as baseline values for evaluating the accuracy of the proposed method. Table 1 gives the theoretical modal parameters of the frame model.

**Figure 2:**
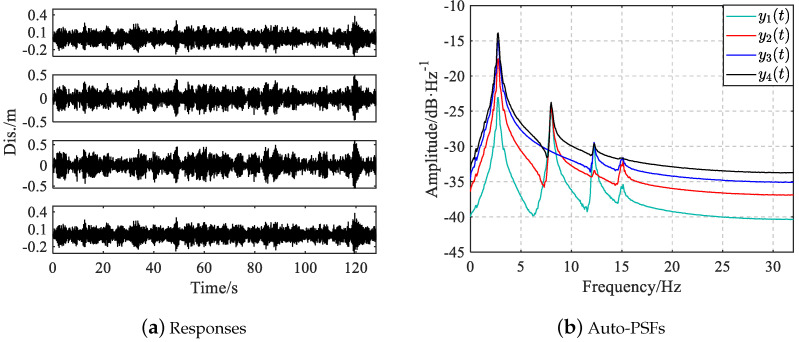
Displacement responses and their auto-PSFs in a typical simulation for the shear frame.

The frame is subjected to uncorrelated zero-mean Gaussian white noise excitation with a standard deviation of 100 N at each story in the horizontal direction. The displacement responses of the frame are computed using Newmark-β method with a constant time step of 0.001 s over 16 s and then resampled with 64 Hz. In addition, the uncorrelated zero-mean Gaussian white noise sequences are added into resampled responses to simulate measurement noise with SNR 20 dB. The full PSD matrix of responses is first calculated using Welch’s method with a Hamming window of 256, 50% overlapping and Fourier transform of 256 data points; the PSD functions are then transformed to correlation functions with inverse Fourier transform; subsequently, the negative part of correlations is set to zero and finally, the PSF functions are derived by applying Fourier transform to reshaped correlations. The responses and its auto-PSFs in a typical simulation are given in Figure 2.

The displacement responses contaminated with measurement noise are used in the AR model to identify the modal parameters of the frame. In addition, the third and fourth columns of the PSF matrix are adopted in the LMF model for the same purpose.

**Figure 3:**
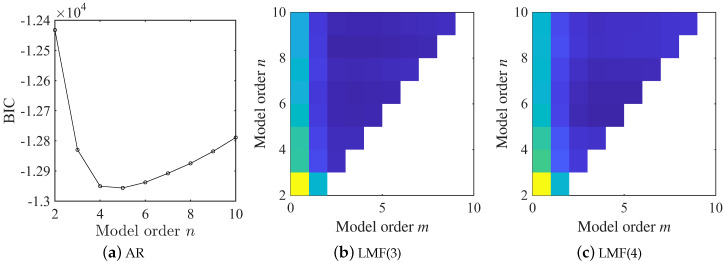
BIC values derived from the AR and LMF models with various mode orders in the shear frame example. $\log_{10}\left(\cdot \right)$ returns the common logarithm of the argument and sign is the sign of the argument; LMF(*i*) represents the LMF model constructed with the *i*th column of PSF matrix.

In this example, the BIC is employed to determine the model orders for both AR and LMF models. As illustrated in Figure 3, the BIC value achieves its minimum value at n=5 for the AR model. In contrast, for the LMF model, no abrupt changes in the BIC value are observed at n=5 and m=3 as the model orders are increased. Consequently, the model order for the AR model is selected as 5, while model orders n=5 and m=3 for the LMF model are determined. The identification results obtained with different methods are finally fused by the method proposed in Section 2.5. Throughout this example, the first component of the mode shape vectors is scaled to unity.

To comprehensively validate the performance of the proposed fusion method, 1000 Monte Carlo simulations are conducted. For each simulation, the modal parameters of the frame model are identified using three methods: the AR model; the LMF models with the third and fourth column of the PSF matrix, respectively; and the proposed fusion method. To implement the fusion method, the variances of modal parameters are computed via the uncertainty quantification approach detailed in Section 2.4. Finally, the Averaged Relative Error (ARE) and sample variance of the identified modal parameters across all 1000 simulations are computed to quantitatively evaluate the performance of the proposed fusion method.

$${\mathrm{ARE}}_{q}^{\left(\gamma \right)}=\frac{1}{N_{\mathrm{MC}}}\sum_{k=1}^{N_{\mathrm{MC}}}\frac{\left|q_{k}^{\left(\gamma \right)}-\tilde{q}\right|}{\tilde{q}},\;{\mathrm{VAR}}_{q}^{\left(\gamma \right)}=\frac{1}{N_{\mathrm{MC}}-1}\sum_{k=1}^{N_{\mathrm{MC}}}{\left(q_{k}^{\left(\gamma \right)}-{\bar{q}}^{\left(\gamma \right)}\right)}^{2}$$

where *q* represents the modal frequency, damping ratio, or a component of a mode shape vector; $q_{k}^{\left(\gamma \right)}$ represents the quantity identified by the method γ for the *k*th simulation; $N_{\mathrm{MC}}$ is the number of Monte Carlo simulations that successfully identify the structural mode corresponding to the quantity *q*; $\tilde{q}$ is the theoretical value of quantity *q*; ${\bar{q}}^{\left(\gamma \right)}$ designates the empirical mean value of $q_{k}^{\left(\gamma \right)}$ over all simulations.

**Figure 4:**
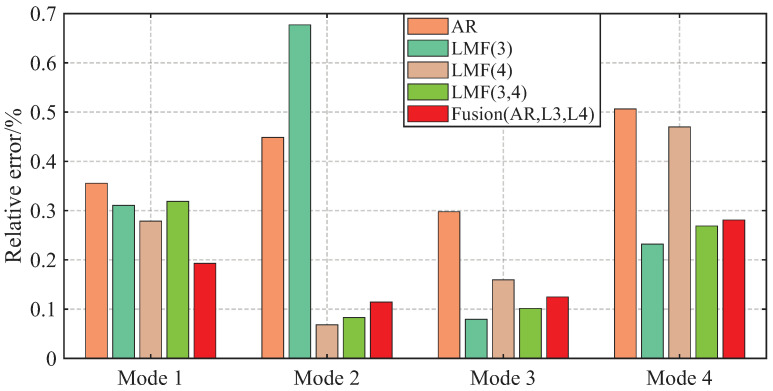
Averaged relative errors of the modal frequencies, identified by different methods over 1000 simulations, for the shear frame.

**Figure 5:**
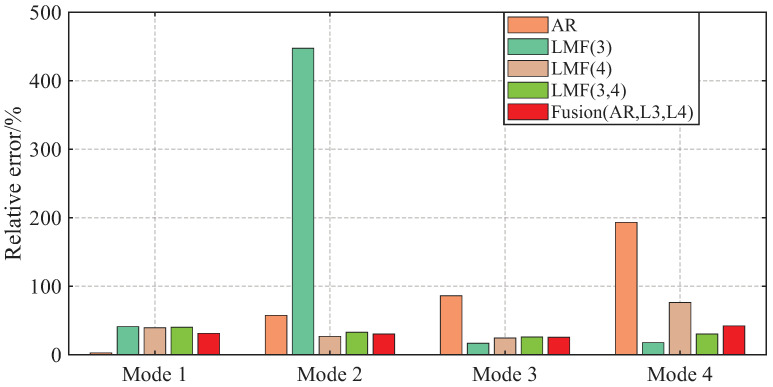
Averaged relative errors of the damping ratios, identified by different methods over 1000 simulations, for the shear frame.

**Figure 6:**
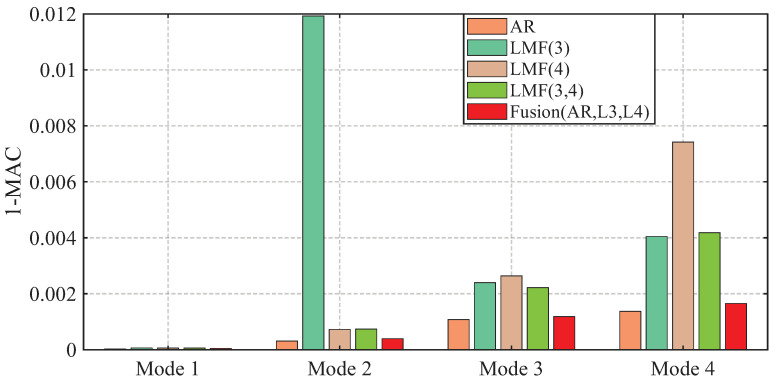
Averaged errors of MAC values between the mode shape vectors identified by different methods and the baseline values for the shear frame.

The AREs of the modal frequencies and damping ratios identified by different methods over 1000 simulations are depicted in Figure 4 and Figure 5. The averaged Modal Assurance Criterion (MAC) values between the identified mode shape vectors and the baseline values are given in Figure 6. For ease of notion, LMF$\left(\cdot \right)$ means that the modal parameters are identified using the LMF model with the specified columns of the PSF matrix, while Fusion(AR,L*i*,L*j*) is the method fusing the results of the AR, LMF$\left(i\right)$, and LMF$\left(j\right)$ models.

The four modal frequencies estimated by all methods are close to the theoretical values. However, the damping ratio estimates are not good, particularly showing very large AREs for the second mode identified by the LMF(3) model, as well as for the third and fourth modes of the AR method. Regarding mode shape vectors, all methods yield satisfactory results with MAC values close to unity, except for the second mode identified by the LMF(3) method.

As demonstrated by the comparative analysis in Figure 4, Figure 5 and Figure 6, the proposed fusion method achieves overall robust estimation for all modal parameters, significantly mitigating the occurrence of extremely large error, although certain individual parameter estimates may not achieve the highest accuracy compared to specialized methods without fusion.

**Figure 7:**
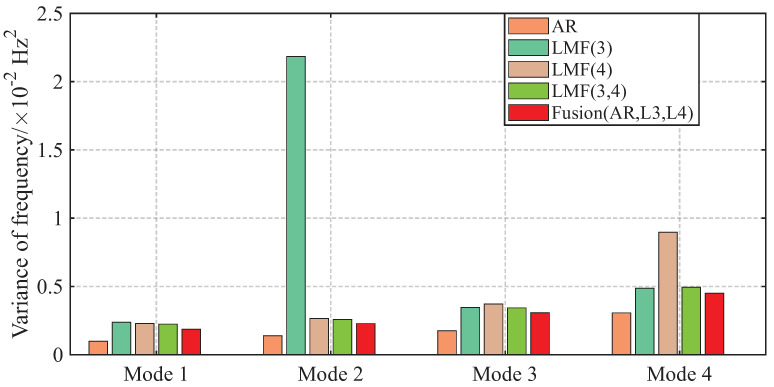
Variances of the modal frequencies identified by different methods over 1000 simulations for the shear frame.

**Figure 8:**
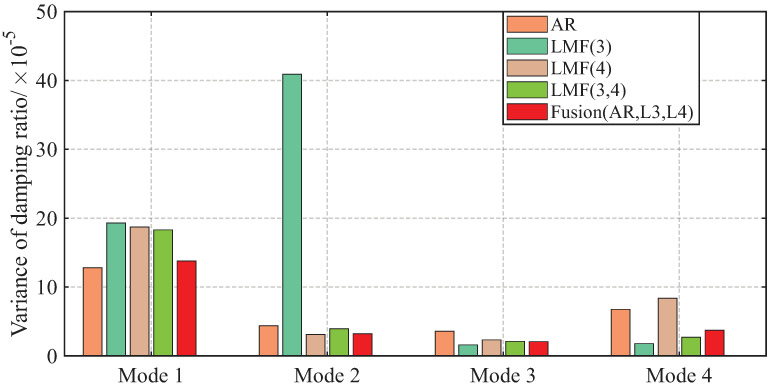
Variances of the damping ratios identified by different methods over 1000 simulations for the shear frame.

**Figure 9:**
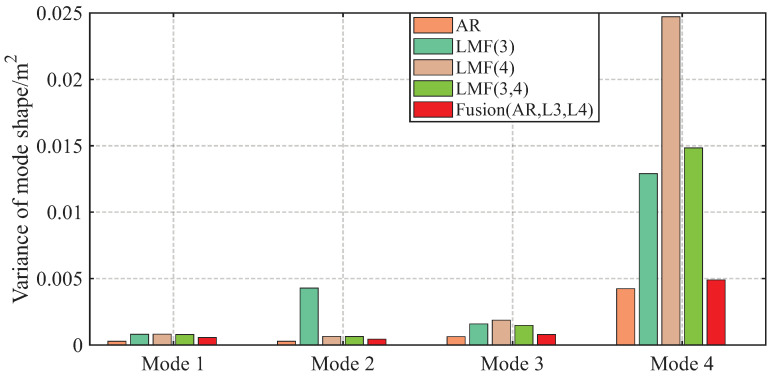
Variances of the second component of the mode shape vectors identified by different methods over 1000 simulations for the shear frame.

The variances of modal parameter estimates over 1000 simulations for the shear frame are compared in Figure 7, Figure 8 and Figure 9, with only the second component of mode shape vectors presented for brevity. The individual methods without fusion exhibit significant variance instability for certain modal parameters: the LMF(3) method for modal frequency $f_{n2}$, damping ratio $\xi_{2}$, and mode shape components $\phi_{22}$ and $\phi_{42}$; the LMF(4) method for $f_{n4}$, $\xi_{4}$ and $\phi_{42}$; and the LMF(3,4) method for $\phi_{42}$. In contrast, Fusion(AR,L3,L4), demonstrates overall enhanced robustness with substantially reduced parameter variances for all modal parameters.

Figure 2b reveals critical challenges regarding structural mode identification: the PSF of $y_{3}\left(t\right)$ lacks distinct spectral peak associated with the second mode, and the fourth peak of $y_{4}\left(t\right)$ exhibits weak energy. Table 2 gives the number of simulations that fail to identify the modal parameters among 1000 simulations for the shear frame. The LMF(3) method presents an 87.3% failure rate in identifying the second mode, and LMF(4) shows an 11.5% missing probability for the fourth mode. This problem can be mitigated by using multiple columns of the PSF matrix in the LMF model. LMF(1,2) and LMF(3,4) achieve 0.3% and 1.8% failure rates for the fourth mode identification, respectively. Notably, two fusion methods, LMF(AR,L1,L2) and LMF(AR,L3,L4), achieve 100% successful mode identification over all 1000 simulations. Therefore, the proposed fusion method can reduce the risk of missing certain structural modes effectively through uncertainty-based identification result integration.

### 3.2. Simply Supported Beam Experiment

**Figure 10:**
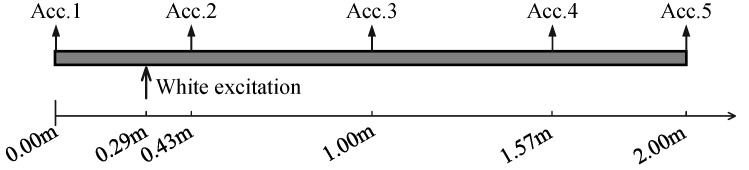
Locations of the excitation and acceleration sensors on the experimental beam.

**Figure 11:**
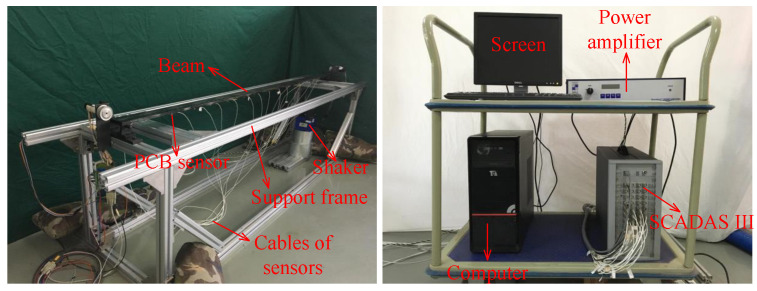
Equipments and real configuration of the experiment.

In this section, the proposed fusion method is applied to an experimental simply supported beam with the following parameters: mass 9.42 kg, length 2.00 m, width 0.06 m and height 0.01 m. An electromagnetic shaker was used to generate a Gaussian random sequence to excite the beam. The acceleration responses under random excitation were recorded using five PCB 333B30 accelerometers and an LMS SCADAS III data acquisition device with a sampling frequency of 256 Hz over 8 s. The sensor placement locations on the beam and real configuration of the experiment are provided in Figure 10 and Figure 11. The properties of the devices used in this experiment are given in Appendix B.

**Figure 12:**
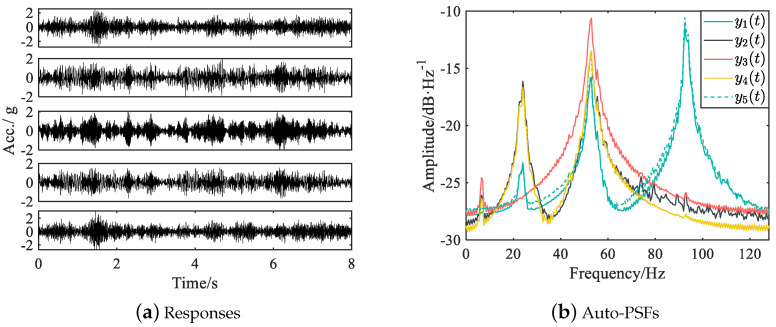
Acceleration responses and their auto-PSFs in a typical test for the experimental beam.

To adequately evaluate the performance of the proposed fusion method, 50 vibration tests were conducted. For each test, the full PSD matrix of recorded responses was computed with a Hamming window of 512, 50% overlap, and Fourier transform of 512 points, and the PSF matrix was subsequently computed. The acceleration responses and the auto-PSFs obtained in a typical test are given in Figure 12. The PSF curves clearly exhibit four peaks within the frequency band of [0, 128] Hz. Therefore, the first four bending modes of the simply supported beam are identified using the recorded acceleration responses and the corresponding PSFs in this experimental example.

**Figure 13:**
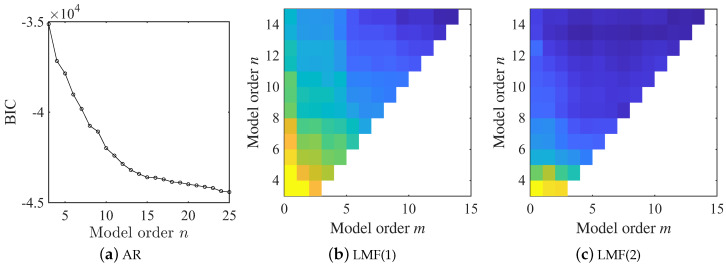
BIC values computed from the AR and LMF models with various model orders in the experiment example.

The model orders of both the AR and LMF models are selected using the BIC criterion, as shown in Figure 13. The combination n=10 and m=5 emerges as a robust choice for all LMF models constructed with different columns of the PSF matrix, since the BIC value indicates no significant variations as model orders continue to increase. However, the BIC value exhibits a continuous descending trend with increasing model order, suggesting that the BIC criterion is less effective in this experimental scenario. This limitation arises because the BIC primarily evaluates model fitness to measured data without incorporating prior physical knowledge, which may be insufficient for real-world applications. To ensure a fair comparison between different methods, the LMF model structure is finally determined as n=10 and m=5, whereas n=10 is adopted for the AR model. Throughout this experimental example, the second component of mode shape vectors is scaled to unity.

**Figure 14:**
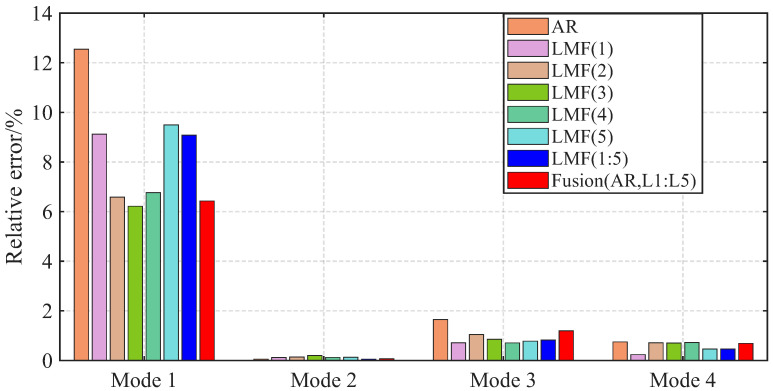
Averaged relative errors of the modal frequencies, identified by different methods over 50 tests, for the experimental beam.

**Figure 15:**
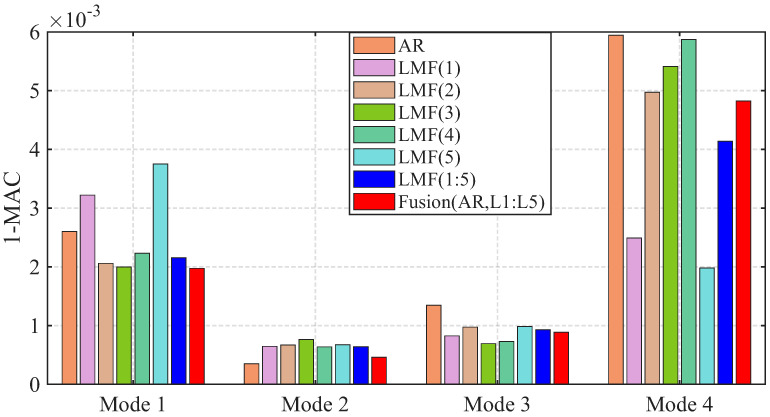
Averaged errors of MAC values between the mode shape vectors, identified by different methods, and the baseline values for the experimental beam.

The modal parameters identified by the well-known Experimental Modal Analysis (EMA) method, i.e., PolyMAX, are used as baseline values. The AREs of modal frequencies identified by different methods are presented in Figure 14. The MAC values of the mode shape vectors are given in Figure 15.

The mode shape vectors identified by different methods agree well with the baseline values with all MAC values close to unity. The first modal frequency estimated by the AR method shows elevated errors compared with other methods. The fusion method Fusion(AR,L1:L5) achieves robust performance for all modal parameters.

**Figure 16:**
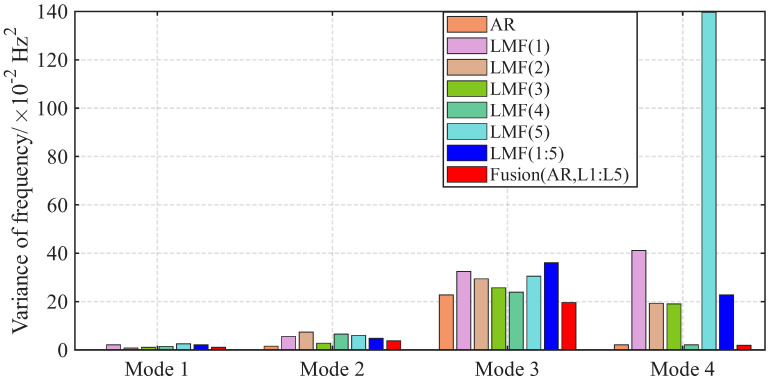
Variances of the modal frequencies identified by different methods over 50 tests for the experimental beam.

**Figure 17:**
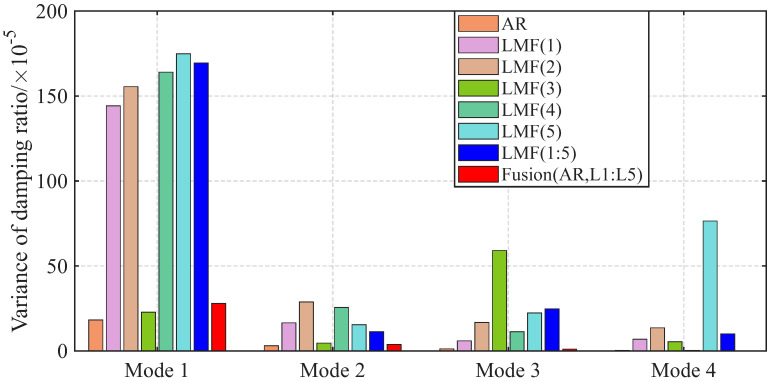
Variances of the damping ratios identified by different methods over 50 tests for the experimental beam.

**Figure 18:**
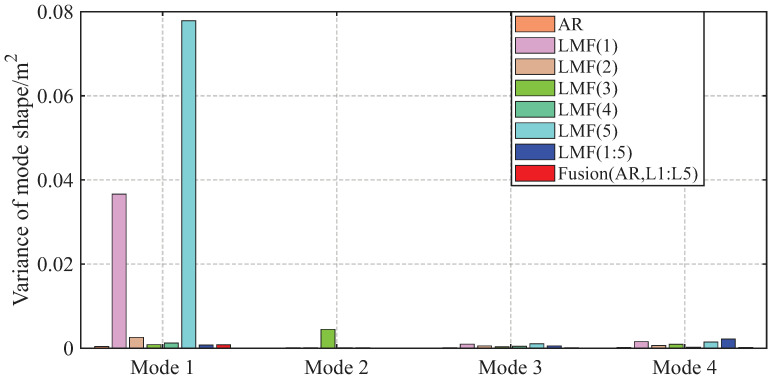
Variances of the first component of the mode shape vectors identified by different methods over 50 tests for the experimental beam.

The variances of identified modal parameters are depicted in Figure 16, Figure 17 and Figure 18. The methods without fusion show significant variances for certain parameters: LMF(1) method for $f_{n4}$ and $\phi_{12}$; LMF(3) for $\xi_{3}$ and $\phi_{22}$; LMF(5) for $f_{n4}$, $\xi_{4}$, $\phi_{12}$; and LMF(1), LMF(2), LMF(4), LMF(5) and LMF(1:5) for $\xi_{1}$. In contrast, the fusion method Fusion(AR,L1:L5) presents robust modal parameter estimates with substantially reduced variances compared with specialized methods.

The number of tests that failed to identify the first mode among 50 tests is listed in Table 3. The AR method exhibits remarkably poor performance, with a failure rate of 96% in identifying the first mode over 50 tests. In contrast, LMF-based methods show considerably better identification capabilities. Both LMF(1) and LMF(5) methods demonstrate 32% failure rates, while the LMF(2) method failed in three tests. In particular, the integration of multiple columns in the LMF model yields substantial improvements, since the LMF(1:2) and LMF(1:5) methods reduce the failure rates to 18% and 4%, respectively. The most significant advancement is observed in the fusion approach. The Fusion(AR,L1:L5) method demonstrates satisfactory identification capabilities, successfully estimating the first mode in all 50 vibration tests. This result again underscores the effectiveness of the proposed fusion method in reducing the risk of missing structural modes.

## 4. Discussion

Section 3 provides two illustrative examples demonstrating the advantages of the proposed fusion method compared to individual approaches without fusion. In particular, the fusion method that merges results from the AR and LMF models with individual columns demonstrates superior performance compared to individual methods. In Appendix A, the computational complexity, specifically associated with the number of required product operations, of various methods is discussed in detail. This analysis demonstrates that the LMF model with a single column is more computationally efficient than its multi-column counterpart. Therefore, an additional advantage of this fusion method lies in its capability to enable parallel computation of single-column LMF models prior to the fusion of results, which could effectively reduce the computational time required for mode identification.

In statistics, both the AIC and BIC criteria are widely used as indicators of model fitness to observed data. In this work, the BIC is used to determine the model structures of the AR and LMF models. However, as illustrated in Figure 13a, the practical implementation of AIC and BIC for model order selection remains challenging, especially for large-scale engineering structures under complex ambient excitation. Furthermore, the hard criteria given in Equation (17) could mitigate but not fully eliminate spurious modes in the identification results for real-world applications. A more effective tool for model order selection and complete spurious mode removal is the Stabilization Diagram (SD). Structural modes are stable with increasing model order, while spurious modes induced by measurement noise or numerical errors display significant deviations. Consequently, structural modes manifest as stable axes in the SD, which can be selected automatically using clustering techniques. This strategy has been extensively applied in Automated OMA (AOMA). Given the substantial computational burden associated with the uncertainty quantification of modal parameters, the model orders of the AR and LMF models can be selected using the SD tool and clustering methods before applying the uncertainty quantification and fusion method presented in this work. Moreover, future integration of the proposed fusion method with AOMA approaches is promising to yield further advancements.

In practical scenarios involving large engineering structures, dynamic responses are often measured using multiple sensor setups due to limitations in sensor availability or data transmission constraints. In such scenarios, modal parameters are typically identified by applying OMA techniques to each setup individually, followed by merging the results. However, the structural modes matching between multiple setups is non-trivial. The mode shapes obtained from multiple setups may not be consistent and certain modes may even be missed in specific setups. The fusion method proposed in this work addresses these challenges by reducing the risk of missing structural modes and providing uncertainty estimates for modal parameters, thereby showing potential to improve the performance of OMA with multiple setups.

The innovation sequences in both AR and LMF models are assumed to follow independent normal distributions, which is the usual situation following the Central Limit Theorem. However, the Gaussian assumption may not hold under specific scenarios where non-Gaussian innovation sequences emerge. In such cases, coefficients and variances cannot be estimated using the NLLF presented in Section 2.3, and the innovation variance may even become undefined under extreme non-Gaussian conditions. Therefore, the data fusion method capable of handling non-Gaussian innovation sequences could be further researched in the future.

## 5. Conclusions

In this study, a fusion method is proposed to identify modal parameters for time-invariant linear structures. A unified model is first presented that combines the parameterized time-domain AR model and frequency-domain LMF models. The model coefficients are estimated using the maximum likelihood method, and the variance matrix is derived from the Hessian matrix of the negative likelihood function. Through the propagation path of the perturbations from the model coefficients to the modal parameters, the variances of the modal parameters are computed utilizing the first-order sensitivity method. Finally, the modal parameters identified from the AR and LMF models are fused with variance-based weights.

A 4-DOF shear frame model and an experimental beam are used to evaluate the performance of the proposed method. Compared with individual methods without fusion, the proposed fusion method exhibits superior performance in obtaining reliable modal parameter estimates, substantially mitigating the occurrence of extremely large estimation errors. Furthermore, the fusion method demonstrates improved identification capabilities, effectively reducing the likelihood of missing structural modes.

## Figures and Tables

**Table 1:** Theoretical modal frequencies and damping ratios of the 4-DOF shear frame model.

Mode	1	2	3	4
Frequency/Hz	2.76	7.96	12.19	14.96
Damping ratio/%	2.97	1.25	1.04	1.00

**Table 2:** Number of simulations that fail to identify the modal parameters among 1000 simulations for the shear frame.

Method	Mode 1	Mode 2	Mode 3	Mode 4
AR	0	0	0	0
LMF(1)	3	0	0	2
LMF(2)	2	0	10	6
LMF(3)	3	873	3	13
LMF(4)	1	0	5	115
LMF(1,2)	0	0	0	3
LMF(3,4)	2	0	2	18
Fusion(AR,L1,L2)	0	0	0	0
Fusion(AR,L3,L4)	0	0	0	0

**Table 3:** Number of tests that failed to identify the first mode among 50 tests for the beam structure.

Method	AR	LMF(1)	LMF(2)	LMF(3)	LMF(4)	LMF(5)	LMF(1,2)	LMF(1:5)	Fusion(AR,L1:L5)
AR	48	16	3	0	0	16	9	2	0

## Data Availability

The data presented in this study are available on request from the corresponding author.

## Abbreviations

The following abbreviations are used in this manuscript:

AIC	Akaike Information Criterion
AOMA	Automated Operational Modal Analysis
AR	AutoRegressive
ARE	Averaged Relative Error
ARMA	AutoRegressive and Moving Average
BIC	Bayesian Information Criterion
CDM	Common Denominator Model
DOF	Degree Of Freedom
LMF	Left-Matrix Fraction
MAC	Modal Assurance Criterion
MLE	Maximum Likelihood Estimate
NID	Normal Independent Distribution
NLLF	Negative Log-Likelihood Function
OMA	Operational Modal Analysis
pLSCF	poly-reference Least Squares Complex Frequency
PSD	Power Spectral Density
PSF	Positive Spectrum Function
RMF	Right-Matrix Fraction
SD	Stabilization Diagram
SNR	Signal-to-Noise Ratio
SSI	Stochastic Subspace Identification
SSI-COV	COVariance-driven SSI
SSI-DATA	DATA-driven SSI
SVD	Singular Value Decomposition
TARMA	Time-dependent AutoRegressive and Moving Average

## Appendix A. Coefficients Estimation for LMF Model for *N*_i_ ≠ 1

### Appendix A.1. Algorithm

The LMF model in Equation (2) can be expressed in a compact form as

(A1) $$G\left[\omega_{k}\right]=-\Theta \Psi \left[\omega_{k}\right]+E\left[\omega_{k}\right],\;\mathrm{vec}\left(E\left[\omega_{k}\right]\right)=\epsilon \left[\omega_{k}\right]∼\mathrm{NID}\left(0,\Sigma_{\epsilon }\right)$$

where $\epsilon =\mathrm{vec}\left(E\right)$ and $\Sigma_{\epsilon }$ is the variance matrix of ϵ and

$$\begin{array}{cc} \Theta & =\left[\begin{array}{cccccc} N_{n_{2}} & \ldots & N_{1} & -L_{n_{3}} & \ldots & -L_{0} \end{array}\right]\\ \Psi \left[\omega_{k}\right] & ={\left[\begin{array}{ccccccc} e^{-in_{2}\omega_{k}T_{s}}G^{T}\left[\omega_{k}\right] & \ldots & e^{-i\omega_{k}T_{s}}G^{T}\left[\omega_{k}\right] & e^{-in_{3}\omega_{k}T_{s}}I_{N_{i}} & \ldots & e^{-i\omega_{k}T_{s}}I_{N_{i}} & I_{N_{i}} \end{array}\right]}^{T} \end{array}$$

where $I_{N_{i}}$ is the unity matrix of dimension $N_{i}\times N_{i}$. The vectorized form of Equation (A1) is

(A2) $$g\left[\omega_{k}\right]=-\left(\Psi^{T}\left[\omega_{k}\right]⊗I_{N_{o}}\right)\theta +\epsilon \left[\omega_{k}\right],\;\epsilon \left[\omega_{k}\right]∼\mathrm{NID}\left(0,\Sigma_{\epsilon }\right)$$

Following the similar derivation process presented in Section 2.2, one can estimate the coefficients and their variance matrix by

(A3) $$\begin{array}{cc} \hat{\theta } & =-{\left(\sum_{k=0}^{N-1}ℜ\left(Q^{H}\left[\omega_{k}\right]Q\left[\omega_{k}\right]\right)\right)}^{-1}\left(\sum_{k=0}^{N-1}ℜ\left(Q^{H}\left[\omega_{k}\right]g\left[\omega_{k}\right]\right)\right)\\ {\hat{\Sigma }}_{\theta } & =\sum_{k=0}^{N-1}ℜ\left(Q^{H}\left[\omega_{k}\right]{\hat{\Sigma }}_{\epsilon }^{-1}Q\left[\omega_{k}\right]\right) \end{array}$$

where $Q\left[\omega_{k}\right]=\Psi^{T}\left[\omega_{k}\right]⊗I_{N_{o}}$ and

$${\hat{\Sigma }}_{\epsilon }=\frac{1}{N}\sum_{k=0}^{N-1}\hat{\epsilon }\left[\omega_{k}\right]{\hat{\epsilon }}^{H}\left[\omega_{k}\right],\;\hat{\epsilon }\left[\omega_{k}\right]=g\left[\omega_{k}\right]+Q\left[\omega_{k}\right]\hat{\theta }$$

### Appendix A.2. Computational Complexity

The dimension of $Q\left[\omega_{k}\right]$ is $N_{i}N_{o}\times N_{o}\left[nN_{o}+\left(m+1\right)N_{i}\right]$. Given the facts that the numerator order *m* is usually smaller than half of the denominator order *n* and $N_{i}\leq N_{o}$, and the data length *N* is relatively large, the number of product operations needed to compute $\hat{\theta }$ or ${\hat{\Sigma }}_{\theta }$ in Equation (A3) is $O\left(n^{2}N_{i}N_{o}^{5}N\right)$, where $O\left(\cdot \right)$ is the big O notation. (Given $A\in R^{d_{1}\times d_{2}}$ and $B\in R^{d_{2}\times d_{3}}$, computing AB requires $d_{1}d_{2}d_{3}$ times of production operations and $d_{1}\left(d_{2}-1\right)d_{3}$ times of summation operations. Since the computational complexity of the summation is much lower than that of the product, the computational complexity of the proposed method is evaluated only considering the production operation.)

In particular, when $N_{i}=1$, the results derived by the coefficient estimation method given in Appendix A are the same as those obtained by the method presented in Section 2.2. However, the computational complexity of the algorithm in Appendix A is much larger, since the Kronecker product operator is used to transform Equation (A1) into Equation (A2). For $N_{i}=1$, the algorithm in Appendix A requires the product $O\left(n^{2}N_{o}^{5}N\right)$ times, while the method presented in Section 2.2 requires only $O\left(n^{2}N_{o}^{2}N\right)$ times. The proposed fusion method consists of multiple steps, including coefficients estimation, uncertainty quantification, and results fusion. The computational complexity in uncertainty quantification for modal parameters is mainly attributed to the term $P{\hat{\Sigma }}_{\theta_{A}}P^{T}$ in Equation (25), which needs the product $O\left(n^{5}N_{o}^{6}\right)$ times, while the computational complexity of the fusion procedure is very partial and could be neglected. The times ofthe product required for modal parameter identification and uncertainty quantification using different methods are compared in Table A1. It is worth noting that, though the computational complexity is assessed using the number of product operations required in the algorithm, the actual computational time for practical implementation would be influenced by various factors, such as code quality, CPU parallelization and technical specifications of the hardware used in practical implementation.

**Table A1:** Times the product is needed for modal parameter identification and uncertainty quantification using different methods.

Method	AR	LMF(*i*)	LMF($1:N_{i}$)	Fusion(AR, $1:N_{i}$)
Number of models	1	1	1	$N_{i}+1$
Coefficients estimation for each model	$O\left(n^{2}N_{o}^{2}N\right)$	$O\left(n^{2}N_{o}^{2}N\right)$	$O\left(n^{2}N_{i}N_{o}^{5}N\right)$	$O\left(n^{2}N_{o}^{2}N\right)$
Variance computation for each model	$O\left(n^{5}N_{o}^{6}\right)$	$O\left(n^{5}N_{o}^{6}\right)$	$O\left(n^{5}N_{o}^{6}\right)$	$O\left(n^{5}N_{o}^{6}\right)$

## Appendix B. Properties of Experimental Devices

The properties of devices used in the simply supported beam experiment are detailed from Table A2, Table A3 and Table A4.

**Table A2:** Properties of PCB 333B30 accelerometers used in the experiment.

Property	Sensitivity	Range	Frequency Range	Resonant Frequency	Weight
Value	100 mV/g	±50 g	0.5 Hz–3000 Hz	≥40 kHz	4 g

**Table A3:** Properties of the ModalShop 2025E electromagnetic shaker used in the experiment.

Property	Peak Sine Force	Stroke Length	Frequency Range	Acceleration (Maximum, Bare Table)	Weight
Value	58 N	18 mm	DC-9000 Hz	72 g pk	6 kg

**Table A4:** Properties of the LMS SCADAS III data acquisition device used in the experiment.

Property	Input Range	ADC	Sampling Rate	Input Channels	SNR
Value	±0.5 mV-±10 V	24 bit	up to 204.8 kHz	32	>90 dB
